# Optical Magnetometry Based on Nanodiamonds with Nitrogen-Vacancy Color Centers

**DOI:** 10.3390/ma12182951

**Published:** 2019-09-11

**Authors:** Adam M. Wojciechowski, Paulina Nakonieczna, Mariusz Mrózek, Krystian Sycz, Andrzej Kruk, Mateusz Ficek, Maciej Głowacki, Robert Bogdanowicz, Wojciech Gawlik

**Affiliations:** 1 Institute of Physics, Jagiellonian University, Łojasiewicza 11, 30-348 Kraków, Poland; 2 Institute of Technology, Pedagogical University of Cracow, Podchorążych 2, 30-084 Kraków, Poland; 3 Department of Metrology and Optoelectronics, Faculty of Electronics, Telecommunications and Informatics, Gdańsk University of Technology, 11/12G. Narutowicza St., 80-233 Gdańsk, Poland

**Keywords:** optical sensor, nitrogen-vacancy, diamond

## Abstract

Nitrogen-vacancy color centers in diamond are a very promising medium for many sensing applications such as magnetometry and thermometry. In this work, we study nanodiamonds deposited from a suspension onto glass substrates. Fluorescence and optically detected magnetic resonance spectra recorded with the dried-out nanodiamond ensembles are presented and a suitable scheme for tracking the magnetic-field value using a continuous poly-crystalline spectrum is introduced. Lastly, we demonstrate a remote-sensing capability of the high-numerical-aperture imaging fiber bundle with nanodiamonds deposited on its end facet.

## 1. Introduction

Recent years have witnessed a substantial increase of interest in studies of color centers in diamond, in particular the nitrogen-vacancy centers. The negatively charged nitrogen-vacancy (NV${}^{-}$) centers in diamond have very interesting optical and spin properties which make them a very good candidate for development of optical/photonic sensors of such quantities as magnetic and electric fields, temperature, and pressure [1,2]. Specifically, NV${}^{-}$ centers (hereinafter we omit the charge sign when referring to the NV${}^{-}$ center) possess non-zero electronic spin (S=1) with long coherence times (>∼3 ms) [3,4] and suitable energy level structure which enable their preparation (spin polarization) and interrogation by visible light and manipulation with radio-frequency/microwave (MW) fields with magnetic resonance techniques. The NV centers permit important applications in magnetometry [2,5], quantum-state engineering [6,7], and quantum metrology [8]. NV diamonds exhibit strong fluorescence and, as elementary carbon samples, they are biologically inert which paves the way to their numerous applications in bio-medical diagnostics [9].

Individual NV centers allow for magnetic surface mapping with nanometric spatial resolution [10,11,12,13,14]. In turn, diamond monocrystals with high concentration ensembles of NV centers offer increased magnetometric sensitivity at the cost of degraded spatial resolution [5,15,16].

Nanodiamonds (NDs) are generally obtained by detonation or crushing larger diamond samples. Their sizes cover wide range between single nano- to micrometers. They are available commercially with preselected sizes and other properties, such as customary surface functionalization. Additionally, substantial density of defects may be introduced to NDs by means of high-energy particle irradiation and subsequent annealing. For example, the ND fluorescence enhancement in the green or red wavelength region may be observed, caused by formation of nitrogen-vacancy-nitrogen (NVN or H3) and NV centers, respectively. Depending on particular applications NDs can be used as powders, suspensions in various liquids (possibly with microfluidic devices), and single NDs deposited in a given location or manipulated with optical tweezers [9,17,18,19].

Many ND applications, such as seeding of substrates for deposition of thin diamond layers via the chemical vapor deposition or preparation of ND-doped polymer nanocomposites and galvanic coatings, require their deposition in a liquid form, hence NDs are often dispersed to form colloidal suspensions [20]. Good understanding of their interactions with the surrounding media is necessary, particularly for biological applications [21]. Dispersing the nanodiamonds in liquid media is also necessary when an ultrasonic disintegration is chosen as a method for de-aggregation of complexes of the diamond particles. Sonication may be also used for decreasing the sizes of NDs since it leads neither to a graphitization of the particle’s surface nor to its contamination with remnants of an abrasive [17]. The colloidal suspensions enable also storing NDs in their de-aggregated form [22]. NDs may be well-dispersed in highly polar solvents such as dimethyl sulfoxide, water and methanol. The colloidal stability and the fluorescence properties of diamond nanoparticles depend on the strength of hydrogen bonds in the suspensions [20,23].

In this work, we present the results of our research on the use of ND powders, which offer an interesting trade-off between sensitivity, resolution and manufacturing costs. We demonstrate the sensing potential of two platforms using NDs with NV centers, i.e., planar layers of dried suspensions and an ND-coated imaging fiber bundle (IFB).

## 2. Experimental Methods and Materials

**Figure 1:**
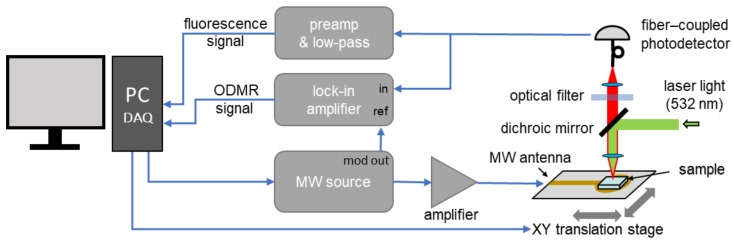
Schematic diagram of the experimental setup. A home-built confocal microscope is used for NV fluorescence detection. ND samples are placed on top of a loop-gap-type antenna and the magnetic resonance is observed for frequencies around 2.9 GHz.

The experimental system is constructed around a confocal fluorescence microscope shown schematically in Figure 1. A moderate-magnification microscope objective (Olympus UPlanFl 10x (Tokyo, Japan), NA = 0.3, WD = 10 mm) is used for focusing the green (532 nm) excitation beam as well as fluorescence light collection. A dichroic mirror (Thorlabs DMLP567, Newton, NJ, USA) and an optical long-pass filter (Thorlabs FEL0600) allow for detection of light with a wavelength in the range of around 600–800 nm using either an avalanche photodiode (APD, Thorlabs APD130A) or a compact optical spectrometer (Thorlabs CCS175). APD signals are filtered and amplified by a preamp (SRS SR560 (Sunnyvale, CA, USA), typical gain: 100) or a lock-in amplifier (SRS SR830).

For optically detected magnetic resonance (ODMR) measurements, a microwave signal from a generator (SRS SG396) is fed through a high-power amplifier (Mini-Circuits ZHL-16W-43+, Brooklyn, NY, USA) and delivered to a loop-gap type antenna structure on a printed circuit board (PCB). The structure is similar to the one described in Reference [24] and is designed to produce a uniform magnetic-field distribution over ∼1 mm distance around its center while maintaining a central hole diameter of 3 mm. ND layers on microscope slides or coverslips are placed directly on the antenna board which is mounted on a motorized translation stage with sub-μm positioning resolution. A rare-earth permanent magnet is placed in several positions nearby the microscope to create a static magnetic field with a varied strength and orientation (direction).

We use two types of ND solutions. The first one is a commercially available 140-nm-size ND suspension in de-ionized water (NDNV140nmHi10ml, Adamas Nanotechnologies Inc., Raleigh, NC, USA). The NDs have a high ∼2–3 ppm NV concentration and their surface is carboxylated to stabilize the suspension. The other suspension is made in-house from a ∼1μm fluorescent ND powder (MDNV1umHi1g, Adamas Nanotechnologies Inc.) and isopropyl alcohol (IPA). The ultrasonic disintegration of the diamonds has been carried out using Bandelin Sonopuls HD 2200 homogenizer (Berlin, Germany). No abrasive has been chosen for the process, allowing particles to be crushed only as a result of collisions between diamonds. The homogenizer has been programmed to work in a pulse mode to avoid overheating of the liquids. A single pulse consisted of 0.5 s of work step and 0.5 s of idle time. The suspension was prepared by 5-min-long high-power ultrasound sonication which disintegrates the ND aggregates while having marginal effect on a mean grain size. In case a smaller particle size is preferred, a longer sonication process, assisted by the addition of zirconium dioxide abrasive beads may be used [25].

## 3. Results

### 3.1. Dried-Out Nanodiamond Layers

**Figure 2:**
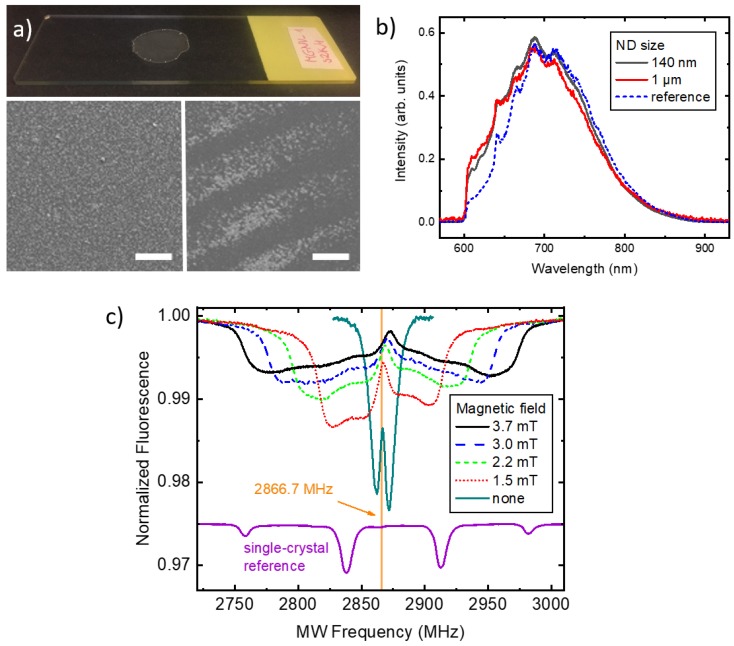
Signals observed with deposited ND ensembles. (**a**) 1-μm-size NDs deposited on a microscope slide (top panel) and SEM images of uniform (left panel) and self-organized (right panel) areas. The scale bar size is 100 μm; (**b**) Optical fluorescence spectra of 140-nm and 1000-nm-size NDs, and a reference bulk type Ib diamond; (**c**) ODMR recorded with a dried 140-nm ND solution as a function of the magnetic-field strength. Vertical line indicates the symmetry frequency for the zero field. The reference signal was recorded with a [111]-oriented single-crystal in a 4 mT field (scaled-down by a factor of 10 and vertically offset for clarity).

The first type of samples we study is formed by depositing droplets of ND solution on a glass substrate, typically a microscope slide or a coverslip (see the top image in Figure 2a). After fully drying out, the solution turns into a layer of deposited NDs. The high resolution images from a scanning electron microscope (SEM) reveal that the residue may have a random ordering with a relatively uniform ND distribution on surface, such as shown in the left panel of Figure 2a, or a self-organized character, depending on the interactions between substrate and the solvent as well as details of the geometry of the laboratory vessels and initial ND concentration in the solution. For example, we have observed coffee-ring type patterns [26], shown in the right panel of Figure 2a, after evaporation of the IPA in a cavity slide covered with a coverslip. In order to prepare uniform and reproducible layers, therefore, certain experience in applying ND solutions to a given type of substrate is needed. On the other hand, the presence of self-organization mechanisms enables creation of novel, interesting structures.

In Figure 2b fluorescence spectra are presented for 140-nm and 1 μm-sized ND layers. Spectra were scaled to the similar area to enable shape comparison. We observe only minor differences in the spectra of both types of particles apart from the overall fluorescence intensity scaling from point to point on both samples. This observation is in contrast to the work presented in [27] where ND size is reflected in the fluorescence spectra, particularly for 5-nm and 50-nm NDs. In here, we use larger NDs which exhibit higher volume-to-surface ratio and, therefore, NVs embedded therein are, on average, less affected by the short-range interactions with the environment.

After confirming that deposited NDs’ optical spectrum is similar to the single-crystalline diamond one we analyze the magnetic resonance spectra. In Figure 2c the ODMR spectrum recorded with 140 nm NDs is shown for several values of the magnetic field. The spectra consist of two branches corresponding to transitions between states $m_{S}=0$ and $m_{S}=\pm 1$. In the zero magnetic field, nearly full degeneracy is observed, characteristic for the NV diamond zero-field splitting of 2870 MHz at room temperature [2,28]. A notable difference is the ∼3 MHz red-shift of the central frequency, which we attribute to the heating effect of the strong (up to 100 mW) laser beam. With the temperature-shift coefficient of 75 kHz/K [28] this frequency change corresponds to the ND temperature increase of about 45 K. Given the small volume of NDs and poor thermal contact with the supporting glass this value is realistic (e.g., a comparable power laser-beam heating rate of ∼10 K/s in a mm-sized diamond plate has been measured in [29]) and shows that sensing applications using ODMR protocol with ND ensembles may require thorough control of thermal effects. On the other hand, the measurement demonstrates potential of the method for local temperature monitoring.

While in zero magnetic field the measured NDs ODMR spectrum did not differ significantly from the single-crystalline one, in non-zero field there are significant differences. The spectra are strongly inhomogeneously broadened by the random orientation of nanocrystals with respect to the direction of the magnetic field [30,31]. This is evident when compared to the reference ODMR signal recorded with a diamond plate with the magnetic field oriented along the [111] crystalographic direction (bottom curve in Figure 2c). In a single-crystal sample, distinct resonances associated with particular spatial NV orientation are well visible. On the other hand, the spectrum of ensemble of NDs is continuous due to the averaging of resonance lines over many possible orientations of NDs with respect to the magnetic-field direction. One of the consequences is that the information on the orientation of the magnetic field vector is lost.

The outermost edges of the spectra correspond to the NDs with the maximal projections of the magnetic field, i.e., those oriented along the field direction. Approximately linear increase of the spectrum width with the field strength is visible in Figure 2c. For larger fields, the nonlinear Zeeman contribution becomes visible and results in the up-shift of the central frequency and the appearance of asymmetry between left and right branches of the spectrum. The overall width of the spectrum is determined by the field strength and, hence, enables its measurement. We discuss a practical method for extracting field value from the ND spectra in the next section.

### 3.2. Magnetic-Field Sensing with ND Ensembles

Magnetometry using single-crystal NV diamonds is already well established [2]. One commonly used magnetometry technique is based on recording the continuous-wave ODMR spectrum and tracking small frequency changes of a particular resonance due to either temperature or the magnetic field. Because of random orientations of NVs with respect to the field vector in poly-crystalline samples and ND suspensions, typical measurement protocols can no longer be directly used with NDs. However, as discussed above and shown in Figure 2c, with a sufficiently large amount of isotropically oriented NDs, the ODMR spectrum becomes effectively averaged over all possible NV orientations and retaining the magnetic field value from the spectrum is possible as has been shown in Reference [30]. There are, however, several caveats limiting the usability of ND layers with this method described below.

In non-uniform samples, such as formed by dried ND suspensions, a spatially varying fluorescence level strongly obstructs the recording of small (∼0.1–1%) ODMR contrast changes between various points on the sample. Additionally, with a poor thermal contact, the ND ensemble can have a wide distribution of temperatures making the analysis of recorded signals even more difficult. One of the ways to alleviate these limitations is the application of amplitude or frequency modulation techniques for the microwave-field and a simultaneous phase-sensitive (lock-in) detection [2,32,33]. Such techniques can be directly used in a confocal setup and also in the wide-field microscopy when a phase-sensitive camera sensor is used [16]. Moreover, the appropriate modulation of two-frequency MW field may be used to distinguish between the temperature and magnetic-field-related resonance shifts [29].

**Figure 3:**
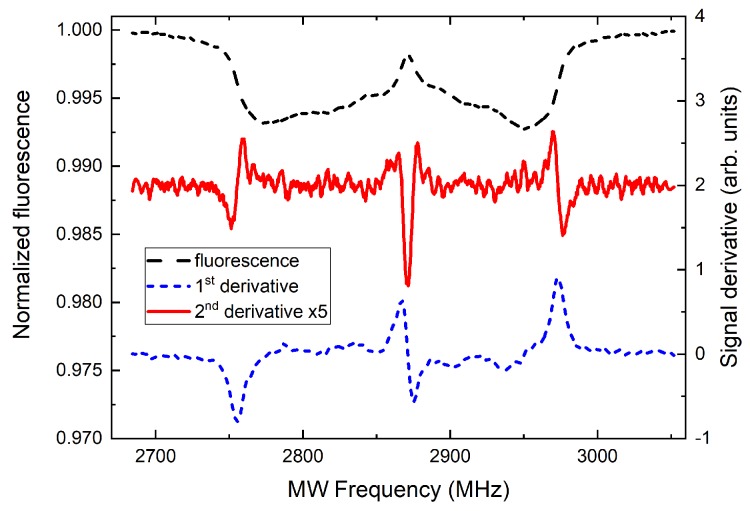
ODMR signals used for magnetometry: spectrum in 3.7 mT field from Figure 2c (dashed black) and its first (dotted blue) and second (solid red, vertically offset) derivatives. Zero-crossings of both the latter signals are independent on the fluorescence intensity and may be used for temperature and magnetic-field sensing.

Figure 3 shows an example spectrum from Figure 2c recorded in 3.7 mT magnetic field (black dashed curve). The spectrum has two wide and continuous resonance branches. The outer edges, as described above, correspond to the classes of NV centers oriented exactly along the field direction. These two groups of NVs have their resonance peaks split due to the magnetic field by $\Delta f=2g\mu_{B}B/h$, i.e., ≈56 MHz/mT, up to several mT where nonlinear Zeeman effect needs to be accounted. The separation of the edges in a continuous spectrum is generally more complicated and given by the combined effect of Zeeman splitting, single-NV-class resonance line-width and the line-pulling effect of other, slightly misoriented NV classes. The situation is even harder if the MW antenna response is not constant across the ODMR spectrum band. Therefore, we propose an approach in which the edge of the spectrum is tracked using lock-in detection. The calculated first and second derivative of the ODMR signal with respect to the frequency are also shown in Figure 3. Such signals can be easily recorded with frequency modulated MW field and a lock-in amplifier (or camera) set to the modulation frequency or its second harmonic. The problem of tracking of the edge position spectrum is therefore simplified to the tracking of the zero-crossing of the lock-in signal (at twice the modulation frequency) which is, to a first degree, insensitive to the recorded fluorescence level. Similarly, the central frequency of the spectrum and, hence, the temperature can be monitored using the zero-crossing of the first derivative of the fluorescence signal.

In order to assess the DC magnetic-field sensitivity, we follow the analysis presented in Reference [2]. We assume MW frequency tuned to the maximal slope of the ODMR signal corresponding to first-derivative peak positions in Figure 3. An infinitesimal magnetic-field value change δB results in the change of the detected fluorescence given by $I_{0}\times \left(\frac{\partial S}{\partial B}\right)\times \delta B\times \Delta t$, where $I_{0}$ is the fluorescence photon rate, *S* is the normalized fluorescence signal, and Δt is the measurement time. If the readout noise is dominated by the photon shot noise, it can be expressed as $\sqrt{I_{0}\times \Delta t}$. This results in the bandwidth-normalized sensitivity η and the minimum detectable field $\delta B_{min}$ given by [2]

(1) $$\eta =\delta B_{min}\sqrt{\Delta t}=\frac{\sqrt{I_{0}}}{I_{0}\times \left(\partial S/\partial B\right)}.$$

Using the Zeeman shift formula, signal slope value of $dS/df\approx 4\times 10^{-4}$ MHz${}^{-1}$ (corresponding to the 3.7 mT signal in Figure 2 and Figure 3), and a conservative photon rate of $I_{0}∼10^{10}$ s${}^{-1}$, we can estimate the current sensitivity to be $\eta \approx 0.9\;\mu T/\sqrt{\mathrm{Hz}}$ around 3.7 mT. Additionally, the sensitivity value increases with increasing field strength due to the reduction of the signal slope dS/df. There is, however, plenty of room for improvement. In particular, the signal contrast may be further enhanced by improving the MW delivery (increasing power) and the fluorescence collection has not been optimized. Finally and most importantly, the overall signal, $I_{0}$, and the resulting sensitivity, η, are strongly dependent on the number (density) of NDs illuminated by the microscope and contributing to the signal.

### 3.3. ND-Coated Imaging Fiber Bundle

**Figure 4:**
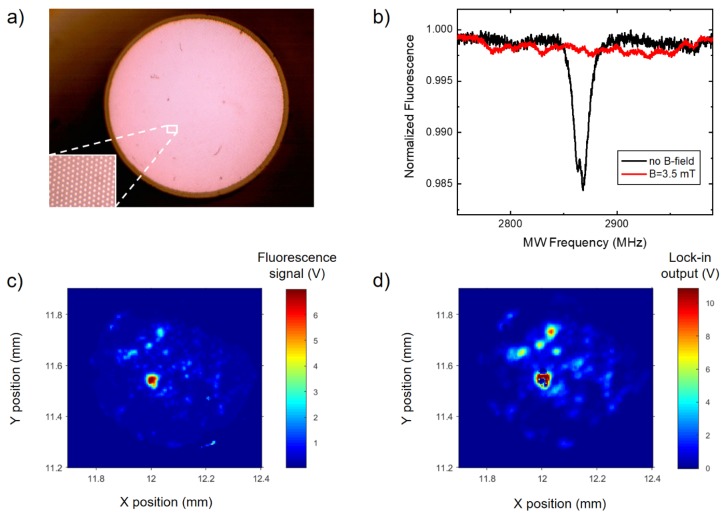
Remote sensing using IFB with a ND-coated distal facet located close to the MW antenna surface. (**a**) Optical microscope image of the fiber bundle. (**b**) ODMR spectrum measured through a single fiber of the IFB in zero and non-zero magnetic fields exhibiting single-crystal type resonances. (**c**,**d**) Fluorescence and the corresponding ODMR contrast maps acquired by scanning the proximal end of IFB.

For practical application of ND layers, we propose a sensing architecture consisting of a high numerical-aperture imaging fiber bundle with one of the facets ND-coated. In a proof-of-concept experiment, we have used a short (length of ~12 mm) section of an IFB. The IFB was fabricated identically to the FB 1–3 samples presented in Reference [34], except for a different drawing speed which resulted in a final diameter of 0.67 mm. It consists of approximately 12,000 individual fibers and its optical microscope image is shown in Figure 4a. Each fiber is made from two types of soft glass, zirconium-silicate ZR3 and borosilicate SK222, with a high contrast between their refractive indices, 1.609 and 1.52 (measured at 589 nm), respectively. This results in a high numerical aperture of NA >0.5 which allows efficient ND fluorescence collection.

One end of IFB was coated by depositing a droplet of the 1-μm ND suspension and allowing IPA to evaporate. It was then mounted in the microscope with a distal (coated) end pointing downward and being located approximately in the MW antenna plane. The microscope focus was set to the proximal IFB end. Figure 4b shows the ODMR spectrum recorded in zero- and non-zero magnetic field with the fluorescence light passing through one of the fibers forming the bundle. Although the length of the IFB in this work is rather short (≈12 mm), it could be easily significantly extended and used for remote imaging techniques, e.g., for endoscopic applications.

A central feature of the IFB is that it enables simultaneous observation of the ND fluorescence over the entire distal-end surface. We have recorded a fluorescence map by scanning the IFB under the microscope and it is shown in Figure 4c. Simultaneously, an ODMR contrast map was acquired in the absence of external magnetic field. This was realized by fixing the MW source frequency at 2865 MHz, i.e., on the maximum of the zero-field resonance visible in Figure 4b (black curve). The source was amplitude modulated (100% depth, 5 kHz modulation frequency) and the lock-in detection was used. Maps of simultaneously acquired fluorescence and ODMR signals confirm that NVs are the actual source of fluorescence. However, in this initial experiment, we have observed very non-uniform deposition of NDs on the fiber surface which precludes the applications we are aiming at. One of the consequences is that the ODMR signal observed through single fibers of the bundle exhibit single-crystalline (discrete) resonance structures, such as shown in Figure 4b (red curve). Nevertheless, these results pave the way for practical applications of ND-coated IFBs. Using smaller (e.g., 140-nm-sized) NDs and appropriate suspension and surface chemistry, a more uniform layer may be deposited. This will enable observation of continuous (averaged over spatial orientations) spectra, such as the ones shown in Figure 2c.

Since the IFB acts in our experiments as a relay of the optical fluorescence signal, its most important parameter is the high numerical aperture, NA >0.5, which stems from the choice of glasses used in fabrication and determines the fluorescence light collection efficiency. The exact shape of the IFB structure determines the achievable spatial resolution and, therefore, should be matched to the application and the rest of the optical setup. On the other hand, the length of the IFB may affect the sensitivity through the attenuation of the optical signal if very long IFB sections (several meters) are used.

## 4. Summary and Conclusions

In summary, we have reported on experiments with dried ND suspensions aiming at the development of photonic sensors with imaging feasibility. With preselected ND sizes and two different solvents we worked towards preparation of future microfluidic applications and/or experiments with optical tweezers with liquid biological samples. In addition to determining static parameters of the samples, we expect the ND ODMR methodology to be helpful in studies of the dynamics of various bio-samples and their constituents moving in a liquid phase.

We deposited dry ND layers containing NV centers on glass structures and found conditions responsible for their various spatial distributions. After illumination with green laser light these layers emitted strong red fluorescence which was recorded with good signal-to-noise ratio by a regular avalanche photodiode detector. Moreover, with proper MW excitation and optical detection, we were able to record magnetic resonance spectra characteristic for the local magnetic field. ODMR spectra of a deposited layer in non-zero magnetic fields exhibit substantial inhomogeneous broadening caused by random orientations of NV spins. While, generally, such broadening reduces magnetic sensitivity, a careful analysis of the broadened spectra may enable accurate determination of the magnetic-field intensity and open the way to magnetic imaging and mapping. In addition, sensing of other modalities, such as temperature and strain, may be possible with NDs [9].

Along with measurements of ODMR signals in NDs deposited on glass, we have also coated the imaging optical fiber bundle [34]. In addition to standard fluorescence imaging through the IFB we performed measurements of the ODMR contrast which resulted in substantial contrast enhancement of individual crystallites of about 1 μm size. As a next step we would like to cover the fiber facet with a uniform ND layer and use the described ODMR imaging for determination of local magnetic field and temperature of the sample. We anticipate the method could be extended to practical endoscopic applications.

In conclusion, several described experiments illustrate the suitability of ND ensembles with high NV concentration for precision magnetic-field sensing. They also demonstrate the significant potential of ND layers as a main part of various photonic imaging sensors.

## Notes

**Sample Availability:** Samples of ND structures are available from the authors upon reasonable request.
